# A meaningful exploration of ofatumumab in refractory NMOSD: a case report

**DOI:** 10.3389/fimmu.2023.1208017

**Published:** 2023-06-28

**Authors:** Yibo Zhan, Min Zhao, Xiaojun Li, Huiying Ouyang, Chenghao Du, Guixian Chen, Zhenzhen Lou, Haoxuan Chen, Yuanqi Zhao, Haoyou Xu

**Affiliations:** ^1^ The Second Clinical College of Guangzhou University of Chinese Medicine, Guangzhou, China; ^2^ State Key Laboratory of Dampness Syndrome of Chinese Medicine, The Second Affiliated Hospital of Guangzhou University of Chinese Medicine, Guangzhou, China; ^3^ Department of Neurology, The Second Affiliated Hospital of Guangzhou University of Chinese Medicine, Guangdong Provincial Hospital of Chinese Medicine, Guangzhou, China

**Keywords:** neuromyelitis optica spectrum disorder, ofatumumab, B cell depletion therapy, relapse, refractory

## Abstract

**Objective:**

To report the case of a patient with refractory neuromyelitis optica spectrum disorder (NMOSD), who, despite showing poor response or intolerance to multiple immunosuppressants, was successfully treated with Ofatumumab.

**Case presentation:**

A 42-year-old female was diagnosed with NMOSD in the first episode of the disease. Despite treatment with intravenous methylprednisolone, immunoglobulin, rituximab and immunoadsorption, together with oral steroids, azathioprine, mycophenolate mofetil and tacrolimus, she underwent various adverse events, such as abnormal liver function, repeated infections, fever, rashes, hemorrhagic shock, etc., and experienced five relapses over the ensuing four years. Finally, clinicians decided to initiate Ofatumumab to control the disease. The patient received 9 doses of Ofatumumab over the next 10 months at customized intervals. Her symptoms were stable and there was no recurrence or any adverse events.

**Conclusion:**

Ofatumumab might serve as an effective and safe alternative for NMOSD patients who are resistant to other current immunotherapies.

## Introduction

Neuromyelitis optica spectrum disorder (NMOSD) is an autoimmune-mediated inflammatory demyelinating disease of the central nervous system, commonly characterized by acute optic neuritis and longitudinally extensive transverse myelitis (1, 2). The aquaporin-4-immunoglobulin G (AQP4-IgG) is highly specific for diagnosis of NMOSD (3). It is typically relapsing, leading to permanent disability in most NMOSD patients (4). A variety of immunotherapies are used for acute treatment and long-term relapse prevention treatment. However, in clinical practice, there are patients with NMOSD who, despite receiving mainstream standardized immunotherapies, still experience multiple relapses due to intolerance or poor response to the therapy. We defined them as patients with some type of refractory NMOSD, although the term “refractory” has not been defined uniformly by now. Treatments of such patients are challenging.

B cells are considered to have a crucial role in the pathogenesis and development of NMOSD (5). More and more attention has been paid to the B cells targeting experimental agents that directly deplete B cells. Ofatumumab (OFA) is a fully human anti-CD20 monoclonal antibody (mAb) attached to a newly epitope. It shows lower allergenicity, lower off-rates and stronger complement-dependent cytotoxicity than the first generation of anti-CD20 monoclonal antibody (eg, rituximab (RTX)) (6). OFA has been approved for the treatment of relapsing multiple sclerosis (RMS) in adults. Theoretically, it can also be used for disease-modifying treatment (DMT) of NMOSD, with scarce reports (7–9) recently. Herein we reported the case of a patient with refractory NMOSD in whom Ofatumumab stabilized her recurrent disease without adverse effects.

## Case description

### First episode

In March 2017, a 42-year-old woman was admitted to the Department of Gastroenterology due to hiccups, acid reflux, vomiting and dizziness for 7 days. She was diagnosed with reflux esophagitis 7 years ago without history of trauma. There was no obvious abnormality in her general blood tests, and the gastroscope suggested chronic superficial gastritis. The clinicians gave her omeprazole, but the symptoms were not significantly relieved. In the following month, she lost more than 10 kilograms and her symptoms worsened. Thus, she was admitted to the Department of Neurology in May 2017.

**Figure 1:**
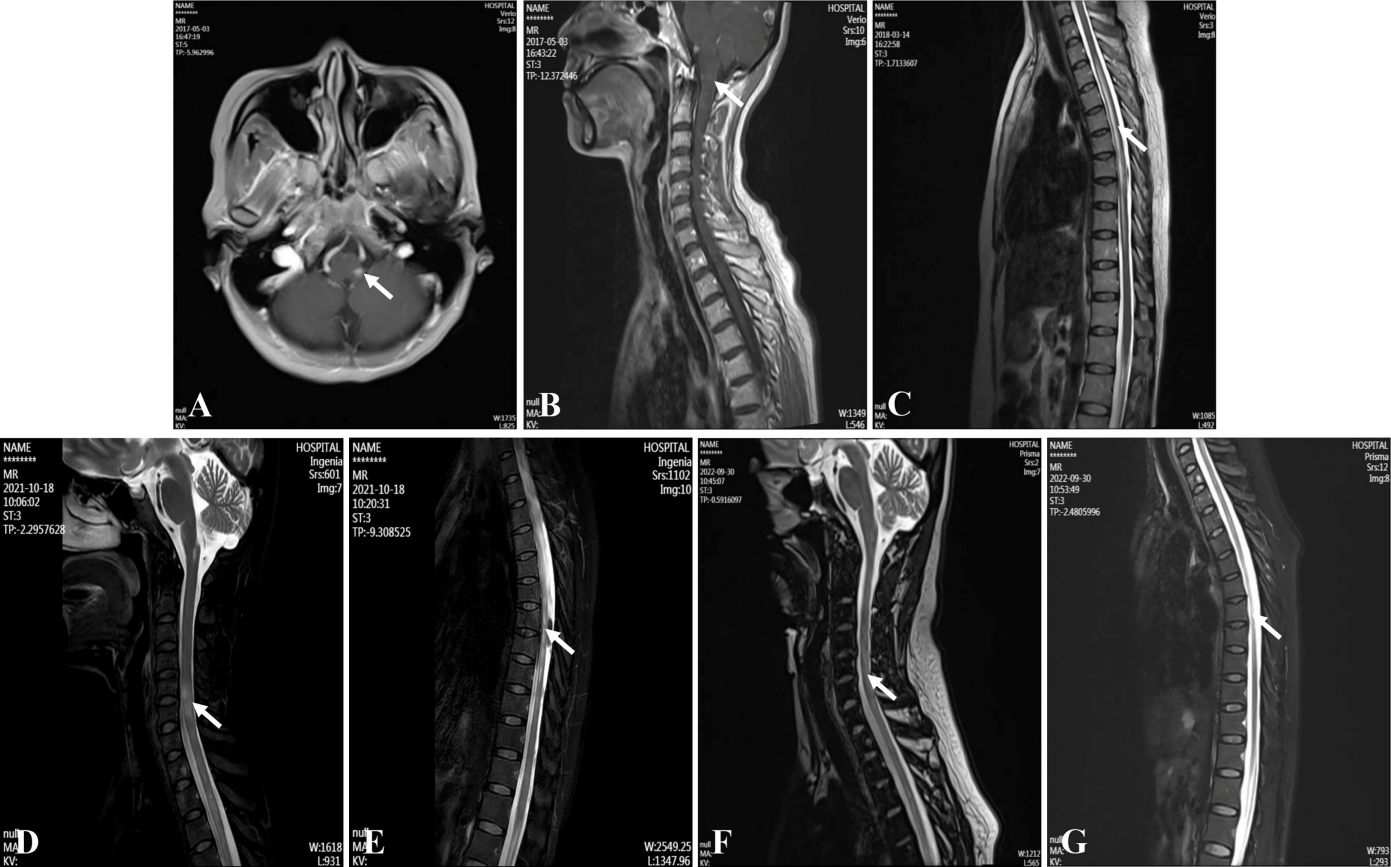
Magnetic resonance imagings of the brain and spinal cord. **(A, B)** Medulla oblongata lesions at the first attack. **(C)** Thoracic cord lesions at the second attack. **(D, E)** Cervical and thoracic cord lesions at the last attack. **(F, G)** Cervical and thoracic cord lesions at four months after using ofatumumab.

On admission, the patient had obvious hiccups, nausea, vomiting, accompanied by persistent dizziness. No obvious abnormality was found in her neurological examination. At noon the next day, she suddenly lost consciousness, accompanied by epileptic convulsions lasting several minutes, which resolved spontaneously without intervention. Blood tests suggested electrolyte disturbances (Na^+^ 114mmol/L, K^+^ 3.3mmol/L, Cl^-^ 84.93mmol/L) and metabolic acidosis (PH=7.132, Lac 14.3mmol/L). She was treated immediately to correct electrolyte disturbances and acidosis. After her symptoms had stabilized, a brain and spinal magnetic resonance imaging (MRI) was performed. It showed abnormal signals in the left posterior part of the medulla oblongata and continuous linear enhancement of the ventral meninges of the cervical spinal cord (**Figures 1A, B**). Cerebrospinal fluid (CSF) test showed high protein (Pro 1009mg/L) and low chloride ion (Cl^-^ 110.65mmol/L). The CSF glucose and white blood cells were in the normal range. According to the MR results, we initially considered it was a NMOSD and AQP4-IgG in cerebrospinal fluid (CSF) was tested. Then, the patient received intravenous methylprednisolone (IVMP) therapy (D1-4 1000mg/d, D5-8 500mg/d, D9-12 250mg/d, D13-15 120mg/d), followed by tapering oral prednisone (60mg/d initially). Meanwhile, the test results showed that AQP4-IgG was positive (CSF titer 1:10, cell-based assay (CBA)) and oligoclonal band test was negative. No obvious abnormalities were found in serum tumor markers, serum immunoglobulin levels (IgA 3.16g/L [1.00-4.20], IgG 11.90g/L [8.60-17.40], IgM 1.16g/L [0.50-2.80]) and other antibodies for autoimmune diseases (such as anti-neutrophil cytoplasmic antibodies, antinuclear antibody and so on). Creatine kinase, rheumatoid factor, anti-streptolysin O, C-reactive protein etc. were within the normal range, and the patient had no joint pain or any abnormality in muscle or skin. The diagnosis of connective tissue disease was also ruled out. The diagnosis of NMOSD was eventually confirmed. Then she started taking azathioprine (AZA) (2mg/kg/d). However, half a month later, she developed abnormal liver function (ALT 105U/L, GGT 63U/L), so the oral AZA was forced to stop. After that, her liver function slowly returned to the normal range.

At discharge, the patient did not have any symptoms of discomfort (Expanded Disability Status Scale (EDSS) score=0). She only maintained oral prednisone (60mg/d). After gradual tapering, she stopped taking prednisone in August 2017. She remained off medication until the second attack.

### Second episode

In March 2018, the patient had sudden urinary retention, constipation and weakness of both lower limbs. She was admitted to our department again. The muscle strength of both lower limbs was graded as 4/5 (Medical Research Council) (EDSS=3.5). MRI showed new abnormal lesions in the spinal cord at T3-T10 levels (**Figure 1C**). She underwent 3-day urinary catheterization and was treated with IVMP (later changed to oral prednisone) and oral AZA (2mg/kg/d). However, she then developed fever, and her fatigue worsened. Urine culture confirmed a urinary tract infection. Therefore, she was treated with intravenous immunoglobulin (IVIG) (20g/d, for 3 days) along with a full course of antibiotics. Unfortunately, the patient developed abnormal liver function again, so she stopped AZA completely and changed her treatment regimen to mycophenolate mofetil (MMF) (1g/d). She stopped taking oral prednisone in July 2018, and only maintained MMF for long-term sequential therapy.

### Subsequent episodes

From April 2020 to March 2021, there were 3 independent recurrences totally, all of which were acute myelitis, and the patient’s symptoms were more serious than the second episode. During the 3^rd^ attack, the muscle strength of her right lower limb dropped to grade 1/5, and she could only walk with the aid of a walker. After treatment with IVIG (20g/d, for 5 days) as well as oral prednisone (60mg/d initially, then on a tapering schedule), she was able to walk slowly on her own and gradually regained her muscle strength. However, approximately 5 months later, she experienced her 4^th^ attack, when her right-lower-limb muscle strength decreased to grade 0/5. The myelin oligodendrocyte glycoprotein (MOG) antibodies and glial fibrillary acidic protein (GFAP) antibodies were both negative in serum and CSF, while the AQP4-IgG positive. Successively treated with IVMP and IVIG, she then suffered from repeated urinary tract infections and even gastrointestinal bleeding and hemorrhagic shock. Because of the adverse events, the clinicians decided to try immunoadsorption. However, after the first trial of immunoadsorption, she had a high fever which made the option of immunoadsorption unavailable. After receiving a long course of antibiotics and rehabilitation treatment, her muscle strength recovered to grade 3/5 at discharge. Unfortunately, about 4 months later, the patient had recurrent weakness of both lower limbs, and the muscle strength both decreased to grade 1/5, which was eventually confirmed as the 5^th^ attack. Like before, she received treatment such as IVIG and antibiotics, and her muscle strength gradually recovered after discharge. What bothered us was that in periods of remission between these attacks, using MMF or tacrolimus still failed to delay the time to relapse.

**Figure 2:**
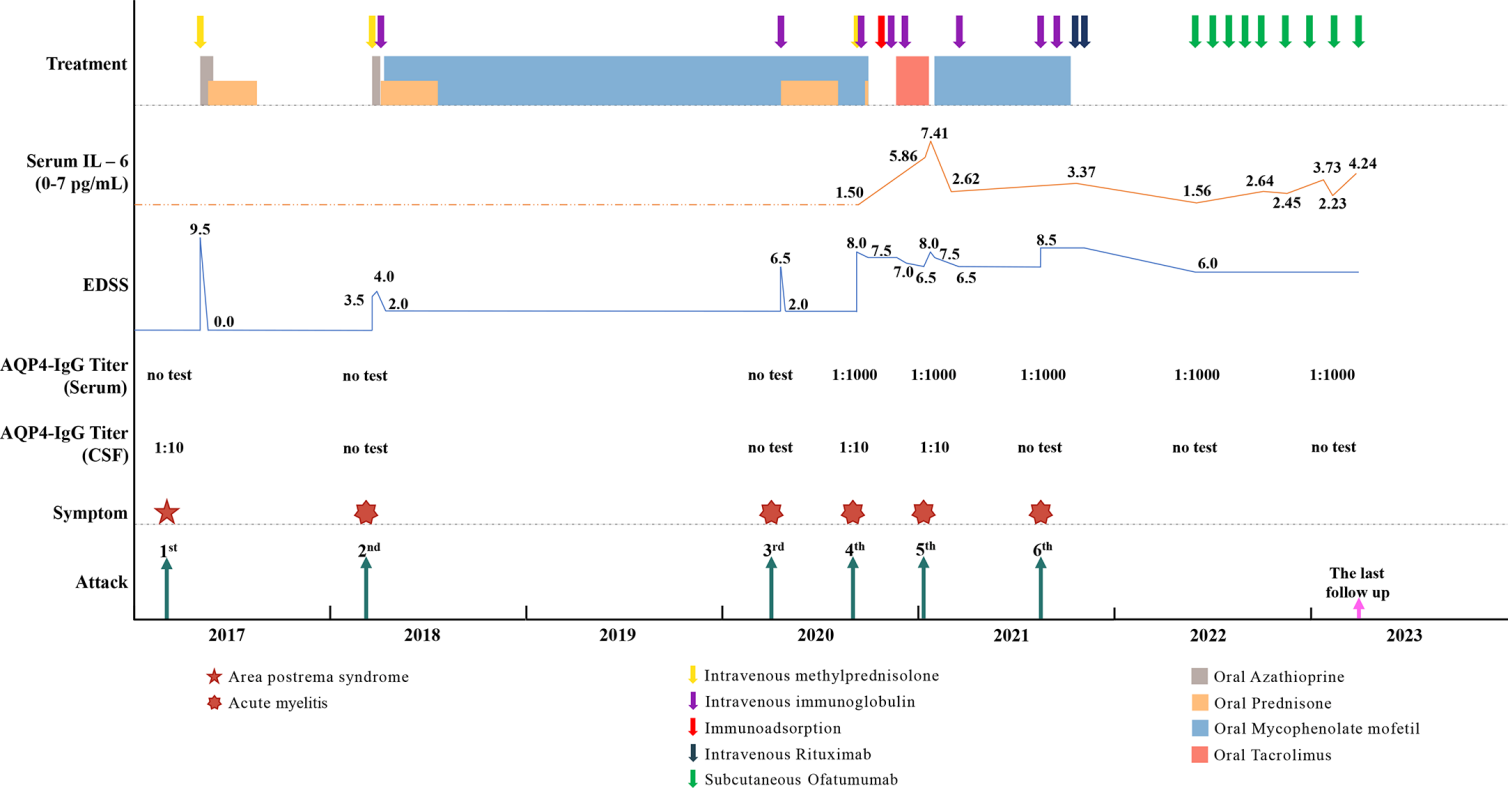
The time points, symptoms, AQP4-IgG titers, EDSS, serum Interleukin-6 (IL-6) level and treatment details of each attack.

In August 2021, the patient suffered the 6^th^ recurrence. On admission, the EDSS score had reached 8.5, and her disability was still progressing (MRI is shown in **Figures 1D, E**). Considering the poor response or intolerance to multiple immunosuppressants, we decided to change the treatment strategy with intravenous RTX. Although the test showed the peripheral CD19+B cells were sufficiently depleted (0.00%) two weeks after the first dose, she still experienced rash all over the body and urinary tract infection, which made RTX impossible for the subsequent treatment. (The time points, symptoms, AQP4-IgG titers, EDSS, serum Interleukin-6 (IL-6) levels and treatment details of each attack are shown in **Figure 2**).

**Figure 3:**
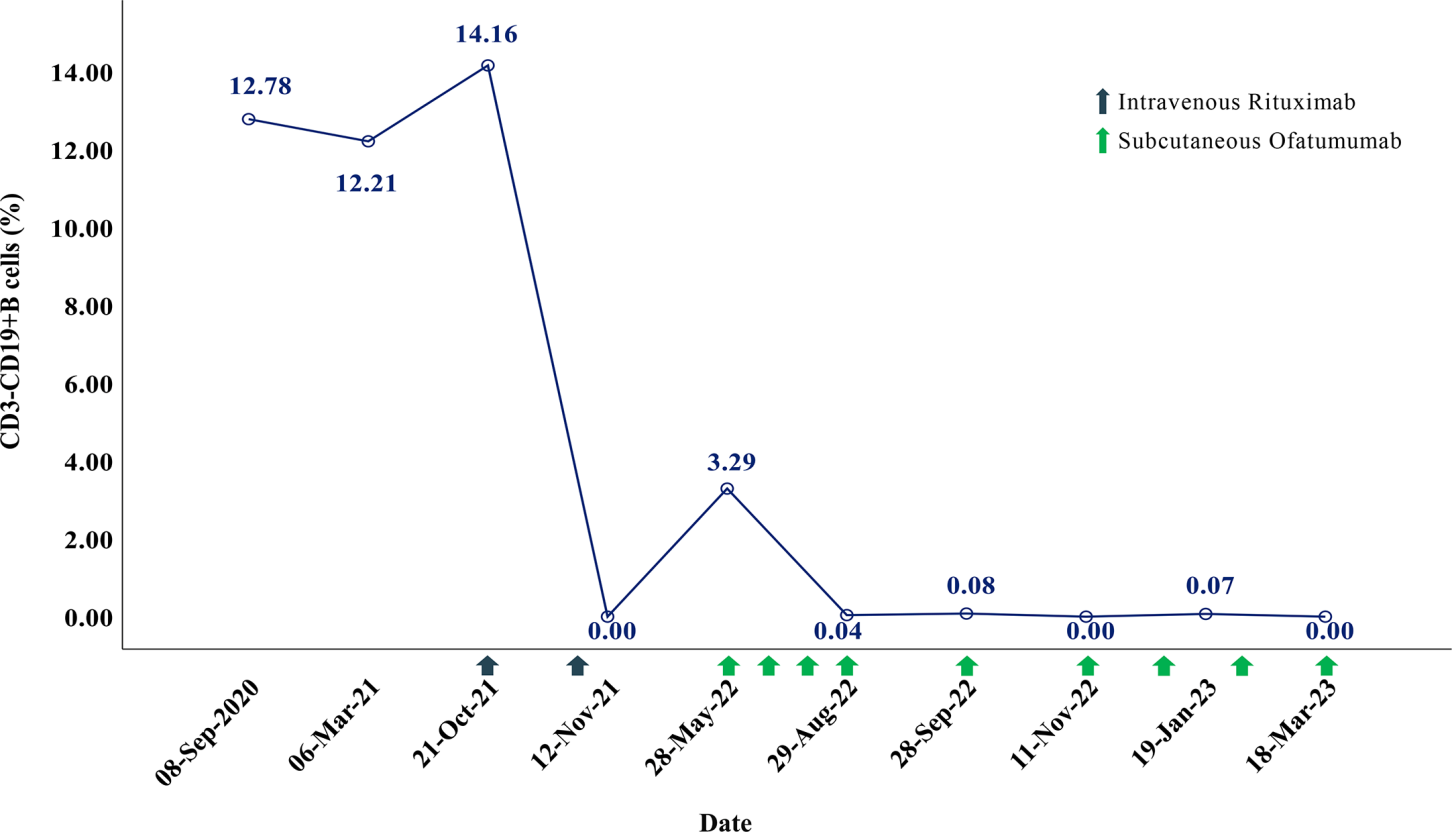
Monitoring of the percentage of CD3-CD19+ B cells.

In May 2022, 6 months after the induction therapy with RTX, the proportion of CD19+B cells recovered to 3.29% (EDSS=6.0). At that time, we noticed that relevant study had shown that OFA had good efficacy in another demyelinating disease, the RMS (10), and OFA had been marketed in China. Considering the patient was intolerant to RTX, we changed the sequential therapy to subcutaneous injection of OFA (20mg q4w, for 4 months). At follow-up after 4 months of OFA treatment, her MRI lesions were significantly reduced (**Figures 1F, G**). We dynamically and closely monitored the levels of CD19+B cells (**Figure 3**).

In view of the fact that previous study had adjusted the usage of anti-CD20 mAbs according to the levels of B cells without reducing the efficacy (11), and dynamic monitoring showed that the CD19+B cells of our patient were fully depleted during the 4 months of OFA treatment, we decided to try to tune the usage of OFA. The dosing interval is then once every 6 weeks. During the treatment with OFA (more than 10 months), the patient’s symptoms were stable without recurrence, the EDSS score remained stable (EDSS=6.0), the proportion of peripheral CD19+B cells was maintained at a very low levels (**Figure 3**), the serum immunoglobulin were maintained at normal levels in the latest follow-up (IgA 2.41g/L [1.00-4.20], IgG 12.10g/L [8.60-17.40], IgM 1.00g/L [0.50-2.80]), and no adverse events occurred.

## Discussion

Herein, we reported the therapeutic effect and safety of OFA in the refractory NMOSD. Our case showed that OFA might have great potential in the treatment of NMOSD.

The use of depleting antibodies (such as RTX and OFA) to target B cells has become an international research hotspot in inflammatory demyelinating diseases (such as multiple sclerosis, NMOSD, etc.) (12, 13). In the stages of B-cell development, the surface molecule CD20 appears at the pre-B-cell stage and is lost at the plasmablast stage. RTX, a chimeric anti-CD20 mAb, depletes CD20+B cells primarily via different mechanisms, such as antibody-dependent cellular cytotoxicity (ADCC) and complement-dependent cytotoxicity (CDC) (14). RTX has shown great efficacy in NMOSD. Two large meta-analyses on NMOSD showed that RTX therapy resulted in a mean 0.79-1.04 reduction in the mean annualized relapse rate (ARR) and a mean 0.44-0.64 reduction in the mean EDSS score. However, adverse effects of the therapy were observed in 26-35% of patients, including infusion-related adverse effects, infection, persistent leukopenia, posterior reversible encephalopathy and even death (15, 16). Given that the intravenous use of B lymphocyte–depleting agents may be limited by infusion-related adverse events, improved drugs are emerging. OFA, a second-generation anti-CD20 mAb, differs from RTX in CD20 binding site. RTX binds to the large extracellular loop of CD20, while OFA binds to the large as well as the small ones. OFA binds to CD20 more tightly and has a slower dissociation rate from CD20 than RTX (17, 18). Thus, it exerts more potent CDC than RTX. In addition, OFA is unique for it is fully humanized and has a modified administration route by subcutaneous injection. Theoretically, it carries a lower risk of adverse reactions and is convenient to use at the same time. In summary, OFA could be a potential new option for patients with NMOSD.

It should be pointed out that the current treatment of NMOSD is still in the stage of exploration, and a unified standard has not yet been formed. Prior to this, there were only a few detailed reports on the treatment of NMOSD with OFA worldwide. The first case (7) reported the efficacy of OFA in refractory pediatric-onset NMOSD with no response to RTX. The second one (8) presented a NMOSD patient that did not respond to RTX and was complicated with hypogammaglobulinemia, who was successfully treated with OFA and IVIG. The third one (9) reported the successful treatment of AQP4-IgG and MOG-IgG double positive NMOSD with OFA. What’s special about our patient is that she tried more immunotherapies than previously reported before she started the OFA injection, including IVMP, IVIG, RTX and immunoadsorption, together with oral steroids, AZA, MMF and tacrolimus. However, she underwent various adverse events, including abnormal liver function, repeated infections, fever, rashes, and even hemorrhagic shock. The recurrence of the disease was not under control, so we decided to use OFA to control her disease during the maintenance phase. We gave her a customized medication rather than the standard treatment.

There is no consensus on which biomarkers to use to monitor the efficacy of B cell depletion therapies (11, 19, 20). In our case report, we used CD19+B cells as the monitoring indicator. At present, the research of the first generation anti-CD20 mAb (RTX) in NMOSD is relatively mature. Previous study has proposed that using B cells to monitor RTX biological efficacy helps reduce the number of administered RTX infusions averagely (11). Prolonging the interval of administration according to the monitoring levels of B cells and carrying out individualized and accurate treatment may also ensure the curative effect. Similarly, we considered that OFA could also be tried in a similar way for it is a second-generation anti-CD20 mAb. In this case, we dynamically monitored CD19+B cells levels and attempted to extend the standard dosing interval, which proved to be safe and effective. We believe that this is a meaningful exploration, which will play an important role in reducing medical costs and side effects caused by the use of drugs. So far, no individualized dose has been reported in other case reports or clinical studies of OFA for NMOSD, which should be further investigated.

The treatment and follow-up of this patient are still going on. The focus of the future treatment is still to dynamically adjust the dosing interval of OFA based on the levels of CD19+ B cells closely monitored in the serum. We will further evaluate the safety and efficacy, and gradually extend this treatment strategy to other NMOSD patients to form exploratory case series or clinical research.

In summary, our case suggests that OFA might serve as an effective and safe alternative for NMOSD patients who are resistant to other current immunotherapies. Large-scale studies are needed for further verification.

## Data availability statement

The raw data supporting the conclusions of this article will be made available by the authors, without undue reservation.

## Ethics statement

The studies involving human participants were reviewed and approved by Ethics Committee of Guangdong Provincial Hospital of Chinese Medicine. The patients/participants provided their written informed consent to participate in this study. Written informed consent was obtained from the participant/patient(s) for the publication of this case report.

## Author contributions

YZ contributed to the study conception, reviewed the literature, drafted the manuscript and drew the figures. MZ, YQZ and HX critically revised the article. XL, HO, CD, GC, ZL and HC analysed and checked the patient’s data. All authors contributed to the article and approved the submitted version.
